# Use of the PiCCO system in critically ill patients with septic shock and acute respiratory distress syndrome: a study protocol for a randomized controlled trial

**DOI:** 10.1186/1745-6215-14-32

**Published:** 2013-02-01

**Authors:** Zhongheng Zhang, Xiao Xu, Min Yao, Huilan Chen, Hongying Ni, Haozhe Fan

**Affiliations:** 1 Department of Critical Care Medicine, Jinhua Municipal Central Hospital, 351 Mingyue Street, Jinhua City, Zhejiang, 3210002, PR China; 2 Department of Surgery, Limb Preservation and Wound Care Research, Boston Medical Center and Boston University School of Medicine, Boston, MA, 02118, USA; 3 Department of Critical Care Medicine, Traditional Chinese Medical Hospital of Jinhua City, Jinhua City, Zhejiang, PR China

**Keywords:** Pulse index Contour Continuous Cardiac Output, Shock, Acute respiratory distress syndrome, Mortality

## Abstract

**Background:**

Hemodynamic monitoring is very important in critically ill patients with shock or acute respiratory distress syndrome(ARDS). The PiCCO (Pulse index Contour Continuous Cardiac Output, Pulsion Medical Systems, Germany) system has been developed and used in critical care settings for several years. However, its impact on clinical outcomes remains unknown.

**Methods/design:**

The study is a randomized controlled multi-center trial. A total of 708 patients with ARDS, septic shock or both will be included from January 2012 to January 2014. Subjects will be randomized to receive PiCCO monitoring or not. Our primary end point is 30-day mortality, and secondary outcome measures include ICU length of stay, days on mechanical ventilation, days of vasoactive agent support, ICU-free survival days during a 30-day period, mechanical-ventilation-free survival days during a 30-day period, and maximum SOFA score during the first 7 days.

**Discussion:**

We investigate whether the use of PiCCO monitoring will improve patient outcomes in critically ill patients with ARDS or septic shock. This will provide additional data on hemodynamic monitoring and help clinicians to make decisions on the use of PiCCO.

**Trial registration:**

http://www.clinicaltrials.gov NCT01526382

## Background

Hemodynamic monitoring and the associated fluid therapy are of critical importance in the management of critically ill patients. The optimization of fluid status remains a challenge in critical care settings because fluid overload will lead to organ edema and an ensuing increase in mortality [1,2], whereas inadequate circulating volume will result in insufficient perfusion pressure and oxygen delivery. Therefore, monitoring the fluid status of critically ill patients is very important. Recent decades have witnessed rapid advances in fluid monitoring techniques. Pulmonary artery catheters have been widely used for more than five decades, but unfortunately their usefulness in improving patient outcomes seems disappointing [3]. The PiCCO system (Pulse index Contour Continuous Cardiac Output, Pulsion Medical Systems, Germany) incorporates a transpulmonary thermodilution technique (TPTD) and continuous pulse contour analysis. It is a minimally invasive technique, which gives beat-by-beat monitoring of cardiac output, and can provide accurate information on volume status and pulmonary edema. Furthermore, the PiCCO monitor is an ‘all-inclusive’ device, which provides a full picture of a patient’s hemodynamic status, including vascular tone, preload, and cardiac function [4]. However, clinical studies investigating the usefulness of the PiCCO system have mainly focused on intermediate physiological parameters, such as fluid responsiveness, oxygenation and pulmonary edema, [5,6], and only a few studies have investigated clinical outcomes of patients managed using a PiCCO monitor [7,8]. Although these studies have promising results, the link between its use and clinical outcomes (for example mortality, length of stay in an ICU, duration of mechanical ventilation) is largely unknown.

Patients with acute respiratory distress syndrome (ARDS) are characterized by increased pulmonary extravascular lung water (EVLW), and may potentially benefit from EVLW monitoring. Simmons *et al*. [9] noticed that a more negative fluid balance in ARDS patients was associated with improved survival. More recently, numerous studies have demonstrated that elevated EVLW is associated with an increased mortality rate [10,11]. A recent meta-analysis by our group also supports this notion [12]. Thus, management algorithms aiming to optimize the extravascular lung water index (EVLWI) are assumed to be beneficial to this group of patients. The other disease entity that is commonly encountered in ICUs is septic shock. This group of patients usually receives infusions of large amounts of fluid during theinitial period to maintain an adequate perfusion pressure. Early goal-directed therapy (EGDT), as proposed by Rivers, is a clinically useful bundle to guide fluid therapy [13]. However, it is not without criticism and the definition of appropriate fluid treatment is still open to debate for this group of patients [14,15].

In this randomized controlled trial, we aim to assess whether the management algorithm using data obtained with a PiCCO system can improve clinical outcomes in critically ill patients with septic shock or ARDS. We hypothesize that a management algorithm based on the PiCCO system will benefit critically ill patients in terms of mortality, length of stay in an ICU and ventilation-free days.

## Methods

The study is designed as a prospective randomized controlled multi-center trial in the ICUs of four tertiary academic centers. These are mixed ICUs treating both surgical and medical patients. The total number of beds is 96. The study will last for a period of 2 years. The end of the study is defined by the last follow-up of the last enrolled patient. The study was approved by the ethics committees of the participating institutions.

### Patient selection

All patients admitted to a participating ICU during the study period will be assessed for potential eligibility. Patients with septic shock, ARDS or both are considered to be eligible. Sepsis is defined as infection plus systemic inflammatory response syndrome (SIRS). General variables are: (1) fever (>38.3°C) or hypothermia (core temperature <36°C); (2) heart rate >90 min^-1^ or >2SD above the normal value for age; (3) tachypnea; (4) altered mental state; (5) significant edema or positive fluid balance (>20 ml.kg^-1^ over 24 hrs); and (6) hyperglycemia (plasma glucose>140 mg.dl^-1^ in the absence of diabetes). Inflammatory variables are: (1) leukocytosis (white blood cell (WBC) count >12000 μL^-1^) or leucopenia (WBC count <4000 μL^-1^); (2) normal WBC count with >10% immature forms; (3) plasma C-reactive protein >2SD above upper normal limit; and (4) plasma procalcitonin >2SD above upper normal limit. Septic shock is defined as a systolic blood pressure (SBP) of <90 mm Hg or mean arterial pressure <70 mm Hg or a SBP decrease >40 mm Hg despite adequate fluid resuscitation [16]. ARDS is defined according to the Berlin definition [17]: (1) the onset should be within one week of a known clinical insult or new/worsening respiratory symptoms; (2) chest imaging shows bilateral opacities that cannot be fully explained by effusions, lobar/lung collapse or nodules; (3) respiratory failure is not fully explained by cardiac failure or fluid overload. An objective assessment may be needed to exclude hydrostatic edema if no risk factor is present. ARDS is divided into three mutually exclusive categories of mild (200<PaO_2_/FiO_2_≤300 with positive end-expiratory pressure (PEEP) or continuous positive airway pressure (CPAP)≥5 cmH_2_O), moderate (100<PaO_2_/FiO_2_≤200 with PEEP ≥5 cmH_2_O) and severe (PaO_2_/FiO_2_≤100 with PEEP ≥5 cmH_2_O). The diagnosis of ARDS is made by a specialist. Chest imaging (for example a chest X-ray or computer tomography) is interpreted by a radiologist. Patients are excluded if they met the exclusion criteria: (1) younger than 18 years; (2) experienced hemorrhagic shock; (3) are moribund, or informed consent cannot be obtained; (4) contraindications to catheter insertion, including overlying infection and arterial grafting; (5) conditions likely to render PiCCO measurements inaccurate, including intracardiac shunts, significant tricuspid regurgitation, and cooling or rewarming [18-20].

### Interventions

#### Experimental intervention

The PiCCO system is used within 2 hours of enrollment. Central venous access is created for the injection of cold water and measurement of central venous pressure (CVP). The choice of type of central venous catheter (CVC) and insertion site are at the discretion of the treating physician. The preferred insertion site is the jugular or subclavian position. We choose the femoral position only when both of these sites are contraindicated. A thermistor-tipped arterial catheter is inserted into the femoral artery. Occasionally, the axillary artery is used when femoral artery catheterization is contraindicated [21]. Then 15 to 20 ml of normal saline at a temperature <8°C is injected into the central vein, and various hemodynamic parameters can be obtained through analysis of variations in blood temperature taken by the temperature sensor of the arterial catheter. At least three cold boluses are required for each calibration to obtain an acceptable precision [22]. The calibration should be performed at least every 8 hours, or following a major change in a patient’s clinical condition [23]. To exclude variations in blood volume and temperature caused by continuous renal replacement therapy (CRRT), the calibration will not be performed immediately after CRRT is switched on or off, and the measurement can be performed after the blood temperature reaches a steady state (after two minutes) [24,25].

**Figure 1:**
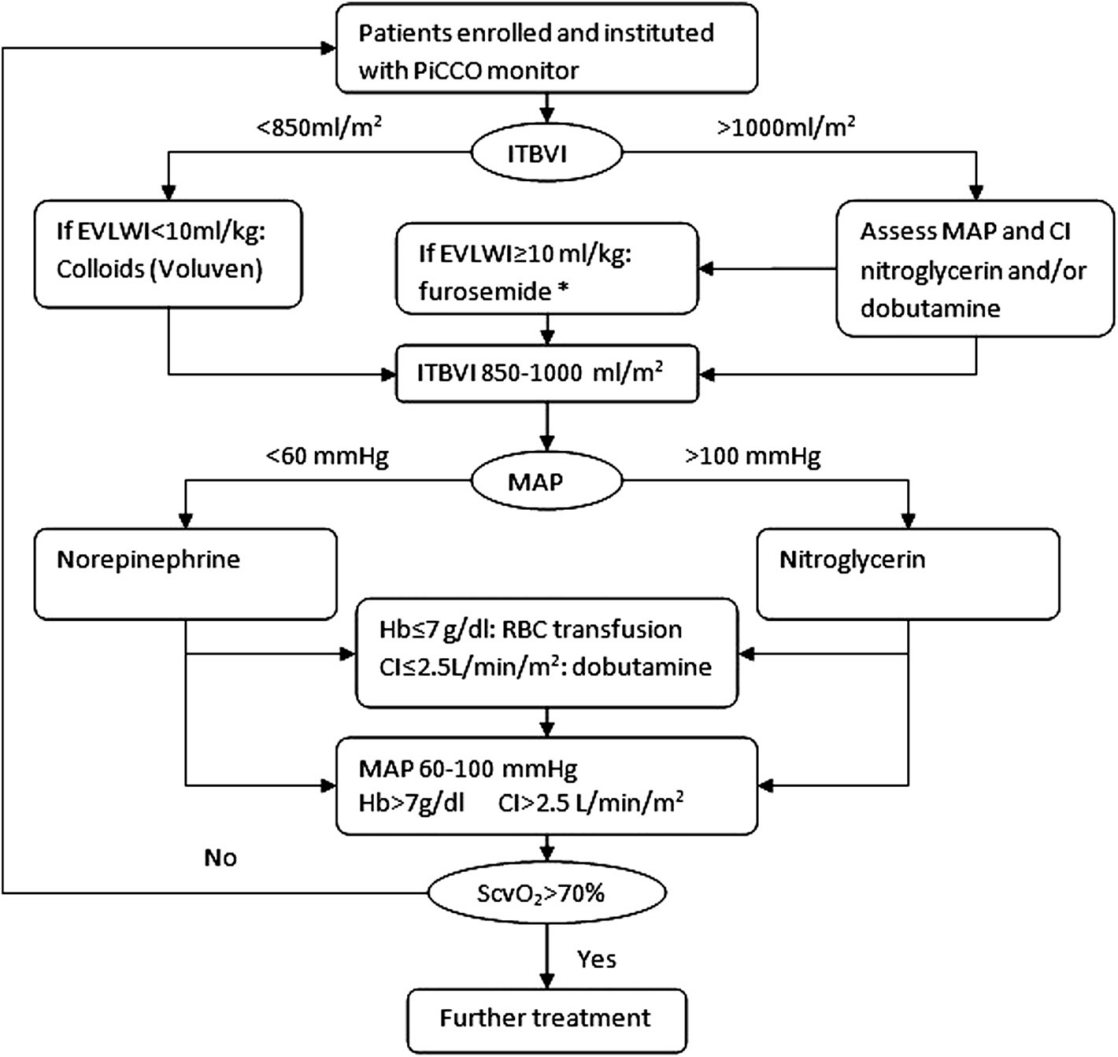
**Algorithm for hemodynamic management according to transpulmonary thermodilution-derived data.** If ITBVI < 850 ml.m^-2^, a 500 ml bolus of hydroxyethyl starch 130/0.4 (Voluven®) was infused over 30 min aiming at an ITBVI of 850 to 1000 ml.m^-2^. The bolus can be repeated if the target is not reached. If ITBVI >1000 ml.m^-2^, nitroglycerin and/or dobutamine are used based on MAP and CI. If EVLWI ≥10 ml/kg, furosemide is used. If MAP < 60 mmHg, norepinephrine is started at 0.05 μg.kg^-1^.min^-1^ with the option to increase at an increment of 0.05 μg.kg^-1^.min^-1^. If MAP > 100 mmHg, nitroglycerin is given at the dose range of 0.5 to 3.0 μg.kg^-1^.min^-1^. RBC transfusion is triggered when Hb <7 g.dl^-1^, and if CI <2.5L.min^-1^.m^-2^ dobutamine is started at the dose of 2.5 μg.kg^-1^.min^-1^. The target is to maintain ScvO_2_>70%. CI, cardiac output; EVLWI, extravascular lung water index; Hb, hemoglobin level; ITBVI, intrathoracic blood volume index; MAP, mean arterial pressure; RBC, red blood cell; ScvO_2_, central venous oxygen saturation.

Fluid management aims to optimize the effective circulating blood volume; vasoactive agents are used to achieve a mean arterial pressure of at least 60 mmHg when the volume status is optimal and extravascular lung water is optimized to a negative fluid balance [26,27]. If the intrathoracic blood volume index (ITBVI) is less than 850 ml.m^-2^, a 500 ml bolus of hydroxyethyl starch 130/0.4 (Voluven®) is infused over 30 min aiming for an ITBVI of 850 to 1000 ml.m^-2^. The bolus can be repeated if the target is not reached. If the ITBVI exceeds 1000 ml.m^-2^, nitroglycerin and/or dobutamine are used based on mean arterial pressure (MAP) and cardiac output (CI). If EVLWI ≥10 ml/kg, furosemide is used. If MAP < 60 mmHg, norepinephrine is started at 0.05 μg.kg^-1^.min^-1^ with the option to increase at an increment of 0.05 μg.kg^-1^.min^-1^. If MAP > 100 mmHg, nitroglycerin is given at the dose range of 0.5 to 3.0 μg.kg^-1^.min^-1^. A red blood cell (RBC) transfusion is triggered when the hemoglobin level Hb <7 g.dl^-1^, and if CI<2.5L.min^-1^.m^-2^ dobutamine is started at the dose of 2.5 μg.kg^-1^.min^-1^. The target is to maintain central venous oxygen saturation ScvO_2_>70% (Figure 1). Dynamic parameters for fluid responsiveness such as pulse pressure variation and stroke volume variation were not included in the protocol due to the requirement for positive ventilation, heavy sedation or paralysis, and a regular cardiac rhythm. The PiCCO system will be removed if the patient is clinically stable for 48 hours as determined by attending physicians. This system can be maintained for a maximum of 10 days. If catheter-related bloodstream infection (CRBSI) is suspected, the central venous catheter will be removed and sent for microbiological study, and the catheter will be exchanged for a new one.

#### Control intervention

**Figure 2:**
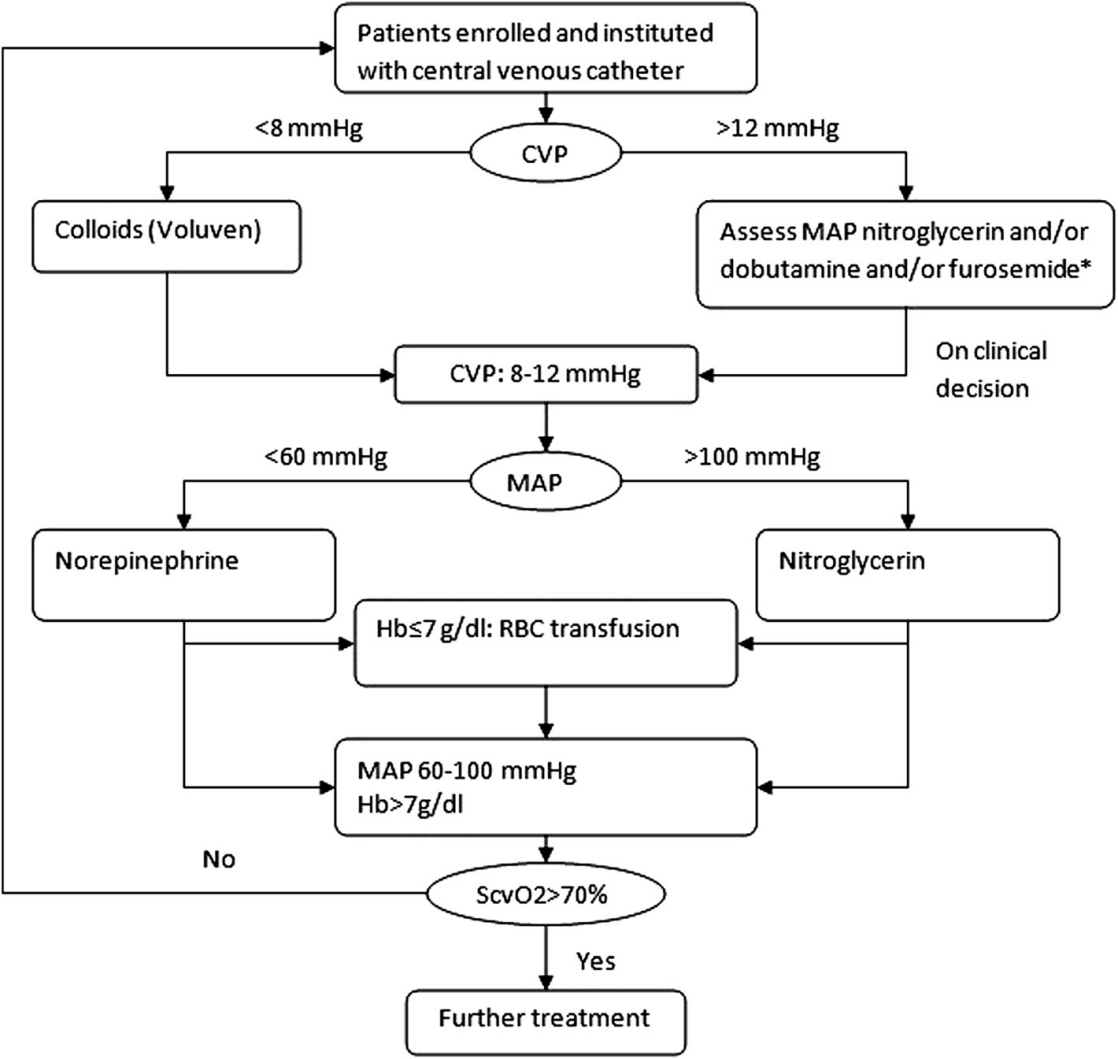
**Algorithm for hemodynamic management in the control arm.** If CVP< 8 mmHg, a 500 ml bolus of hydroxyethyl starch 130/0.4 (Voluven®) was infused over 30 min aiming at a CVP of 8 to 12 mmHg. The bolus can be repeated if the target is not reached. If CVP >12 mmHg, furosemide and/or nitroglycerin and/or dobutamine are used at the discretion of the attending physician. If MAP < 60 mmHg, norepinephrine is started at 0.05 μg.kg^-1^.min^-1^ with the option to increase at an increment of 0.05 μg.kg^-1^.min^-1^. If MAP > 100 mmHg, nitroglycerin is given at the dose range of 0.5 to 3.0 μg.kg^-1^.min^-1^. An RBC transfusion is triggered when Hb<7 g.dl^-1^. ScvO_2_ is maintained >70%. CVP, central venous pressure; Hb, hemoglobin; RBC, red blood cell; ScvO_2_, central venous oxygen saturation.

Patients in the control arm will not receive PiCCO monitoring, but a central venous catheter is routinely inserted. If the CVP is less than 8 mmHg, a 500 ml bolus of hydroxyethyl starch 130/0.4 (Voluven®) is infused over 30 min aiming to give a CVP of 8 to 12 mmHg. The bolus can be repeated if the target is not reached. If the CVP exceeds 12 mmHg, furosemide and/or nitroglycerin and/or dobutamine are used at the discretion of the attending physician. If MAP is less than 60 mmHg, norepinephrine is started at 0.05 μg.kg^-1^.min^-1^ with the option to increase at an increment of 0.05 μg.kg^-1^.min^-1^. If MAP > 100 mmHg, nitroglycerin is given at the dose range of 0.5 to 3.0 μg.kg^-1^.min^-1^. An RBC transfusion is triggered when Hb<7 g.dl^-1^. ScvO_2_ is maintained >70% (Figure 2).

#### Study end point

The primary endpoint is 30-day mortality (death from any cause before day 30).

#### Secondary outcome measures

ICU length of stay: Since the time of ICU discharge may be affected by the availability of beds on a general ward, we predefine the ICU length of stay as the day from ICU admission to the day when the patient is ready for ICU discharge. A patient is considered ready for discharge when he or she is no longer in need for vital organ support.

Days on mechanical ventilation: The criteria for termination of mechanical ventilation: a cooperative patient, recovery from primary disease, hemodynamically stable, adequate and strong cough reflex, positive end-expiratory pressure <5 cmH_2_O, pressure support <10 cmH_2_O and the spontaneous breathing trial is successfully passed.

Ventilator-free days during 30-day period.

Days of vasoactive agent support: The sum of the number of days with one or more vasoactive agents to maintain a mean arterial pressure >60 mmHg.

Vasoactive-agent-free days in 30-day period.

ICU-free survival days during 30-day period.

Maximum sequential organ failure assessment (SOFA) score during the first 7 days.

#### Data safety monitoring board

A Data Safety Monitoring Board chaired by the director of the Department of Critical Care Medicine (XX) of Jinhua Municipal Central Hospital, comprising experts in clinical trials, biostatistics and intensive care, has been established. The board will review data on patient characteristics, compliance and study outcome in an interim analysis (based on the availability of primary outcomes for 350 patients). The study will be terminated if one arm turns out to be harmful compared to the other.

Adverse events were considered to be: hematoma, pneumothorax, arterial emboli, catheter-relatedbloodstream infection, hemorrhage, pseudoaneurysm or arrhythmia.

#### Sample size and statistical analysis

We assumed that the overall mortality at 30 days is 40% [28,29]. A sample size was calculated to detect a 10% difference in mortality at day 30 between the two groups with a two-tailed test, a significance level of 5% and a power of 80%. We plan to include a total of 708 subjects.

Baseline characteristics will be reported. The difference in 30-day mortality between the two groups will be tested using a chi-square test and intention-to-treat (ITT) analysis. Time-to-event variables including length of stay in the ICU, duration of mechanical ventilation and 30-day survival are compared between the two groups using a log-rank test. Multivariate analysis (the Cox proportional hazards regression model) is used to estimate the hazard ratio adjusted for age, severity of illness and disease category. Age and the APACHE II score will be managed as continuous variables. Adverse events will be reported according to the ITT protocol. All tests are two sided and *P*<0.05 is considered to be statistically significant. Statistical analysis will be performed using Stata 11.0 (College Station, TX 77845, USA).

#### Randomization

Eligible consecutive patients will be randomly assigned to one of the treatment groups using randomization sequences generated by computer and stratified according to the primary diagnostic category on admission, namely, septic shock and ARDS. Allocation concealment is achieved using sequentially numbered, sealed opaque envelopes.

#### Blindness

The blinding of non-pharmacological treatment in our study is complicated and costly. However, every effort is made to mask the study researchers and participants [30]. We use the same electrocardiogram (ECG) monitor (Philips IntelliVue Patient Monitor with a PiCCO module) for both intervention and control arms. A sham procedure of injecting cold water is performed for patients in the control arm. Investigators who collect baseline characteristics and follow-up participants are blinded to patient assignment.

#### Ethical aspects

The study has been approved by the ethics committees of Jinhua Municipal Central Hospital, the Traditional Chinese Medical Hospital of Jinhua City, Dongyang People’s Hospital and the First People’s Hospital of Yongkang City. The research will be explained in detail to the patient or the next-of-kin prior to enrollment. The explanation will include the type and method of the study, the complications of PiCCO monitoring and the potential benefit or harm of the intervention. Written informed consent will be obtained from the patients or their surrogates. The patient or surrogate can withdraw from the study at any time.

The study is conducted according to the Declaration of Helsinki.

## Discussion

Fluid therapy is an art in the treatment of critically ill patients. To facilitate treatment and improve patient outcomes, every effort has been made to enhance the technology used for measuring relevant physiological parameters. These parameters allow us to better understand the underlying mechanisms of certain disorders. For instance, in septic shock, hemodynamic monitoring will typically present a low systemic vascular resistance and high or normal-high cardiac output. Intuitively, an understanding of pathophysiological mechanisms will eventually translate into improvements in clinical outcomes. Pulmonary artery catheters (PACs) have been widely used for several decades only because they allow clinicians to get more information regarding hemodynamic status, but without clinical evidence of improved outcomes. In response to this lack of evidence, several randomized controlled trials have been conducted to test the usefulness of PACs in improving clinical outcomes [31-33]. Unfortunately, all these trials consistently show that PACs do no better than controls regarding patient outcomes, but they increase medical costs significantly. These disappointing results have tempered the enthusiasm for PACs, and a survey showed that the use of PACs decreased by 65% during recent decades [34-36].

However, the failure of PACs in improving clinical outcomes does not mean that the measurement of hemodynamic parameters is useless. On the contrary, it reflects the limited understanding of the complex hemodynamic characteristics in critically ill patients. With the declining use of PACs, there is an increasing number of alternatives for hemodynamic monitoring. The PiCCO system is one such alternative, which integrates a wide series of both static and dynamic hemodynamic parameters through a combination of TPTD and pulse contour analysis. The PiCCO system has several advantages over a PAC. First, PiCCO is less invasive than a PAC, so that the severe complications attributable to PACs, such as a pulmonary embolism, pulmonary artery rupture and arrhythmia, are less likely to occur [37]. Furthermore, the arterial canalization required for PiCCO is safe and no significant adverse events for the usually used femoral site have been demonstrated [38,39]. Second, the PiCCO system has the unique ability to measure global end diastolic volume (GEDV) and EVLW. Multiple studies have demonstrated the superiority of GEDV over filling pressures (for example the central venous pressure and pulmonary artery wedge pressure) in estimating cardiac preload [40-42]. EVLWI is a quantitative assessment of pulmonary edema, which has been shown to be associated with clinical outcomes, and EVLW-directed fluid therapy may be potentially useful in improving patient clinical outcomes, such as the duration of mechanical ventilation, length of stay in ICU and mortality [43-45]. Third, PiCCO allows continuous measurement of cardiac output, which is potentially useful given the rapidly changing hemodynamic conditions in critically ill patients. Fourth, the PiCCO system is an ‘all inclusive’ hemodynamic monitor, which integrates an array of parameters. Adjusting fluid parameters by considering all aspects of cardiac performance may confer better clinical outcomes than using a single hemodynamic parameter [4]. However, compared with a PAC, the PiCCO device cannot monitor pulmonary artery (PA) pressure, pulmonary artery occlusion pressure (PAOP) and mixed venous oxygen saturation. Filling pressures, as previously mentioned, are not an accurate parameter of cardiac preload. Thus, PAOP measurements are not mandatory and can be replaced by GEDV to estimate cardiac preload. Furthermore, although not numerically exchangeable, mixed venous oxygen saturation can be approximated by central venous oxygenation saturation [46-48]. Therefore, based on current knowledge, the PiCCO system appears to be superior to the PAC in hemodynamic monitoring of critically ill patients.

The current study aims to investigate the usefulness of the PiCCO system in improving outcomes for patient with ARDS and septic shock. We believe that this randomized controlled trial will provide new evidence for fluid management in critical care settings.

## Trial status

The trial is currently recruiting study subjects.

## Abbreviations

ARDS: acute respiratory distress syndrome; CI: cardiac output; CRBSI: catheter-related bloodstream infection; CRRT: continuous renal replacement therapy; CVP: central venous pressure; ECG: electrocardiogram; EGDT: early goal-directed therapy; EVLW: extravascular lung water; EVLWI: extravascular lung water index; GEDV: global end diastolic volume; Hb: hemoglobin level; ITBVI: intrathoracic blood volume index; ITT: intention to treat; MAP: mean arterial pressure; PA: pulmonary artery; PAC: pulmonary artery catheter; PAOP: pulmonary artery occlusion pressure; PEEP: positive end-expiratory pressure; PiCCO: Pulse index Continuous Cardiac Output; RBC: red blood cell; SBP: systolic blood pressure; ScvO_2_: central venous oxygen saturation; SOFA: sequential organ failure assessment; TPTD: transpulmonary thermodilution technique; WBC: white blood cell.

## Competing interests

The authors declare that they have no competing interests. None of the authors received financial funds from Pulsion Medical Systems.

## Authors’ contributions

ZZ and XX contributed equally to this study and they coordinate, enrol and monitor the study subjects. HN has contributed to the study protocol. HF enrolls subjects and collects data. He is also responsible for monitoring safety. HC and ZZ contributed to the study protocol and they are responsible for the performance of PiCCO monitoring. MY contributed to the revision of the manuscript and acts as statistical consultant. All authors read and approved the final manuscript.
